# Evaluation of the Transport and Binding of Dopamine-Loaded PLGA Nanoparticles for the Treatment of Parkinson’s Disease Using In Vitro Model Systems

**DOI:** 10.3390/pharmaceutics16050571

**Published:** 2024-04-23

**Authors:** Karin Danz, Jana Fleddermann, Marcus Koch, Elena Fecioru, Lorenz Maahs, Nicole Kinsinger, Johannes Krämer, Annette Kraegeloh, Sylvia Wagner

**Affiliations:** 1 Fraunhofer Institute for Biomedical Engineering IBMT, Joseph-von-Fraunhofer-Weg 1, 66280 Sulzbach, Germany; 2 INM—Leibniz Institute for New Materials, Campus D2 2, 66123 Saarbrücken, Germanymarcus.koch@leibniz-inm.de (M.K.); annette.kraegeloh@leibniz-inm.de (A.K.); 3 Eurofins PHAST Development GmbH & Co. KG, Byk-Gulden-Str. 2, 78467 Konstanz, Germany; 4 Pharmacelsus GmbH, Science Park 2, 66123 Saarbrücken, Germany; maahs@pharmacelsus.de (L.M.); kinsinger@pharmacelsus.de (N.K.); 5 Disso e.K., Preussenstr. 15, 66424 Homburg, Germany; johanneskraemer@disso-science.com

**Keywords:** nanoparticles, blood–brain barrier, transport study, in vitro models, Parkinson’s disease

## Abstract

The treatment of Parkinson’s disease has been moving into the focus of pharmaceutical development. Yet, the necessity for reliable model systems in the development phase has made research challenging and in vivo models necessary. We have established reliable, reproducible in vitro model systems to evaluate the binding and transport of dopamine-loaded PLGA nanoparticles for the treatment of Parkinson’s disease and put the results in context with comparable in vivo results. The in vitro models have provided similar results concerning the usability of the investigated nanoparticles as the previously used in vivo models and thus provide a good alternative in line with the 3R principles in pharmaceutical research.

## 1. Introduction

The treatment of neurodegenerative diseases is an ever-increasing point of focus in pharmaceutical development. Parkinson’s disease (PD), specifically, has been showing increasing incidence levels [1,2]. Therefore, the discovery of new or the improvement of existing treatment options is vital [3,4]. The underlying cause of the widely known and most prevalent motoric symptoms of PD stems from an imbalance in neurotransmitters due to the loss of dopaminergic neurons in the substantia nigra [5]. Substituting the lost dopamine to re-establish the balance in neurotransmitters, and thus mitigate the motoric symptoms has long been the aim in the treatment of PD. The biggest challenge in this context is the blood–brain barrier (BBB) [6]. Due to its highly selective nature, it largely restricts the substances circulating in the systemic vessels from reaching the brain parenchyma and makes the treatment of PD and other neurodegenerative diseases a difficult undertaking. When considering dopamine replacement in the context of PD treatment, the dopamine precursor L-Dopa has been successfully used for years, as it is capable of crossing the BBB, but this therapy has also not been without challenges [7,8]. The direct substitution of dopamine appears to be the most promising option, but dopamine is incapable of crossing the BBB by itself, and an increased dopamine concentration in the peripheral blood vessels may cause many undesirable systemic side effects. That is why systemic dopamine is rapidly metabolised by the ubiquitous dopa decarboxylase. An alternate method for transporting dopamine across the BBB while simultaneously shielding it to prevent side effects and metabolisation is needed. Nanoparticles appear to be the obvious solution in this context and have been widely discussed in the treatment of neurodegenerative diseases [9,10]. In this context, poly(lactic-co-glycolic) acid (PLGA) is a very desirable base polymer for the production of nanoparticles, as it is both biocompatible and biodegradable, and nanoparticles based on it can be tailored in their chemical properties, degradation speed and uptake routes and are very versatile in terms of their usable surface modifications and the substances that can be incorporated [11,12]. Methods for the encapsulation of dopamine have been investigated [13,14,15] and discussed as treatment options and investigated in in vivo mouse models with promising success [13]. Yet, one of the largest obstacles is successfully relating the results of such in vivo experiments to the expected effect in humans. Additionally, in accordance with the 3R principles of animal experiments—replacement, reduction and refinement—it is necessary to consider whether the testing of new nanoparticle systems in in vitro cell models would produce similar, and perhaps more reliably transferable, results as the animal experiments commonly in use today. The quality of the used model systems is essential in this context and should thus be carefully considered.

In nanoparticle studies of the blood–brain barrier, cell lines and primary cells of human and non-human origin are widely used [16,17]. While these models are well able to mimic the main characteristics of the BBB as related to protein expression and permeability, the results still translate poorly to the human in vivo situation [18]. To achieve the best possible correlation between in vitro and in vivo results, it is not only essential to use the best possible model system but also to take into account the possible reactions of the prepared nanoparticles in the human vascular system. In terms of in vitro model systems, cell models based on human stem-cell-derived BBB cells have emerged as the most promising approach. These cell models show a good correlation in terms of physico-chemical properties, protein expression and transport behaviour with the in vivo situation, as has been shown by a number of different research groups [19,20,21]. Considering the nanoparticles and the vascular system, nanoparticles for crossing the BBB must be targeted specifically towards this purpose. Covalently linking peptides, proteins or other substances with specific transporters present in the BBB to the nanoparticle surface is a widely used practice [22,23]. Some of these potential surface proteins with specific transporters in the BBB are apolipoproteins [24]. But directly linking these proteins to the particle surface is not necessary. It has been shown that specific coatings for nanoparticles are capable of attracting apolipoproteins from among those circulating freely in the blood [25,26]. Therefore, it might be a better strategy to allow the nanoparticles to recruit the necessary proteins for recognition at and uptake by the BBB from the plasma of the subject they are administered to. Therefore, this study investigates the viability of delivering dopamine across an advanced in vitro model of the BBB by encapsulating it in biodegradable nanoparticles and using a simple surface coating to target the BBB. Additionally, the validity of the used stem-cell-based in vitro model is analysed.

## 2. Materials and Methods

### 2.1. Nanoparticle Synthesis and Characterization

#### 2.1.1. Nanoparticle Synthesis

Dopamine-loaded nanoparticles were synthesized based on the protocol by Pahuja et al. [13], with some modifications [14]. A quantity of 100 mg dopamine hydrochloride (SigmaAldrich, Taufkirchen, Germany) was dissolved in 1 mL of 10 mM hydrochloric acid (VWR International GmbH, Darmstadt, Germany) with 2% PVA (SigmaAldrich, Taufkirchen, Germany). This first aqueous phase was added dropwise to the organic phase, consisting of 50 mg PLGA Resomer 502H (SigmaAldrich, Taufkirchen, Germany) dissolved in 5 mL dichloromethane (SigmaAldrich, Taufkirchen, Germany) with 250 µL 1 mg mL^−1^ Lumogen Red 305 (BASF SE, Ludwigshafen, Germany) dissolved in DMSO, while being homogenised by a Sonopuls (Bandelin electronic, Berlin, Germany) for 1.5 min at 30% pulse strength. The second aqueous phase consisting of a 1% PVA solution in water was stirred at 1200 rpm on a magnetic stirrer. After the first homogenisation was complete, 10 mL of the second aqueous phase were added dropwise to the initial emulsion while being sonicated at 70% pulse strength for 1.5 min followed by an additional 3.5 min at 30% pulse strength. This emulsion was then added dropwise to the remaining second aqueous phase under constant stirring at 1200 rpm. The emulsion was stirred for at least 2 h at room temperature to enable solvent evaporation, followed by standing for 30 min in a desiccator. The remaining nanoparticle suspension was centrifugated for 45 min at 4 °C and 10,000× *g*. The supernatant was removed with a pipette and the nanoparticle pellet resuspended in deionised water under agitation at 700 rpm and 4 °C. The nanoparticle suspension was centrifuged and resuspended in deionised water once more. An aliquot of the resulting nanoparticle suspension was used for the initial analytical characterisation, and the rest was lyophilised for further use.

#### 2.1.2. Lyophilisation of Nanoparticles

The lyophilisation was performed by freeze-drying the nanoparticle suspension using an alpha 1–4 LSCplus lyophilisator (Martin Christ, Osterode, Germany) based on the protocol by Kleimann [27]. After gravimetric analysis, the nanoparticle suspension was diluted to a nanoparticle concentration of 4 mg mL^−1^. Equal amounts of the diluted nanoparticle suspension and a 6% poloxamer 188 solution were added to lyophilisation vials and lyophilised using the following cycle: The initial drying was achieved at −40 °C at a pressure of 1.0 bar for 24 h followed by a second drying step at −20 °C at 0.01 bar for 12 h or until all remaining water had been removed. The lyophilisation vials were sealed to preserve the vacuum and stored in the dark at 4 °C until needed.

#### 2.1.3. Water Content Determination

A combined Karl Fischer (KF) oven and coulometric titration method was used for the water content determination in the samples [28]. A Karl Fischer automated 874 Oven Sample Processor connected to a KF 851 Titrando coulometer with generator electrode without diaphragm (Metrohm AG, Filderstadt, Germany) was used. Approximatively 30 mg of the sample was weighed in a 10 mL glass vial and closed immediately. All samples were prepared in triplicate in addition to the appropriate controls.

#### 2.1.4. Particle Characterisation

For the initial characterisation before lyophilisation, the nanoparticle suspension was diluted 1:100 in deionised water and the hydrodynamic diameter, polydispersity index (PDI) and zeta potential were determined using the zetasizer ZS (Malvern Pananalytical, Worcestershire, UK) with three independent measurement cycles, each consisting of ten measurements per sample.

After lyophilisation and reconstitution of the particles, the primary particle size, size distribution and morphology were determined by transmission electron microscopy (TEM). For this purpose, particle lyophilisates were freshly reconstituted in water. The diluted particle dispersions were rinsed over a holey-carbon-coated copper grid (type S147-4; Plano, Wetzlar, Germany), air dried and imaged by TEM (JEOL JEM-2100 LaB6; JEOL, Tokyo, Japan) at a 200 kV accelerating voltage. Micrographs of 1024 × 1024 pixels were acquired using an Orius SC1000 CCD camera (Gatan, Pleasanton, CA, USA) with 2× binning and an acquisition time of 0.5 s. The primary particle sizes were calculated using GIMP software (Version 2.10.22). Here, TEM images from three independent sample preparations and measurements were manually analysed with regard to the particle diameter. In total, a particle number of n = 110 for particles loaded with dopamine and dye and n = 93 for particles without dopamine and only with dye were investigated. The mean primary particle size and standard deviation were finally calculated. Nanoparticle tracking analysis [29] (NTA; NanoSight LM10, Malvern Panalytical, Worcestershire, UK) was used to determine the average hydrodynamic particle diameter and size distribution in water. Measurements using diluted particle dispersions (1:100 in water) were performed at room temperature. Particles were added to the measuring unit and illuminated by applying a 405 nm laser light. The scattered light was captured using a camera over multiple frames. Each sample was measured five times (5 × 60 s video). The average hydrodynamic particle diameter was determined from the resulting number size distribution profile. The zeta potential of the reconstituted particles was measured with a Zetasizer NanoZSP (Malvern Panalytical, Worcestershire, UK) at 150 V, using 0.01 M KCl as the background electrolyte.

#### 2.1.5. Nanoparticle Leaching

The leaching of Lumogen Red 305 dye from particles was analysed by fluorescence measurements using the Infinite M 200 PRO plate reader from Tecan (Männedorf, Switzerland). Fluorescence at 610 nm was measured by applying an excitation wavelength of 580 nm. Particles were freshly reconstituted and diluted 1:50 in PBS. The fluorescence of diluted particles (100 µL per well) was measured in 96-well plates to obtain the control values (100%). To determine the leaching of dye molecules, particles were ultrafiltered (after 0 h or after 24 h at room temperature) through modified polyether sulfone membranes (molecular weight cut-off = 100 kDa; Pall, Dreieich, Germany) by centrifugation (12,000× *g*, 10 min). The fluorescence of the filtrate was measured and given as a % of the control value.

### 2.2. Cell Culture

Cell culture media and solutions were purchased from Fisher Scientific (Schwerte, Germany) unless stated otherwise. The hiPS cell line Ukki011-A was obtained from the European Bank for induced pluripotent stem cells (EBiSC) [30] and is also registered on https://hpscreg.eu, last accessed on 25 January 2024.

#### 2.2.1. Cell Lines

bEnd.3 cells were cultivated in DMEM with 10% foetal bovine serum (FCS), 100 U mL^−1^ penicillin and 100 µg mL^−1^ streptomycin.

hBMECs were cultivated in RPMI 1640 with 20% FCS, 100 U mL^−1^ penicillin, 100 µg mL^−1^ streptomycin, 2 mM GlutaMAX, 1 mM sodium pyruvate, 1% non-essential amino acids and 1% MEM vitamin solution.

LUHMES (LGC Standards, Wesel, Germany) were cultivated and differentiated as described by Scholz et al. [31] in flasks pre-coated with 50 µg mL^−1^ poly-L-ornithine and 1 µg mL^−1^ fibronectin with proliferation medium (Adv. DMEM/F12 with 1% N2-Supplement, 2 mM L-glutamine and 40 ng mL^−1^ basic fibroblast growth factor) or differentiation medium (Adv. DMEM/F12 with 1% N2-supplement, 2 mM L-glutamine, 1 mM dibutyryl cAMP, 2 ng mL^−1^ GDNF and 1 µg mL^−1^ tetracycline), depending on their intended status as proliferating or differentiated cells.

#### 2.2.2. hiPSC-Derived Human Brain Capillary Endothelial Cells

The human brain capillary endothelial cells (hBCECs) used in the transport studies were differentiated from human induced pluripotent stem cells (hiPSCs) as described by Stebbins et al. [32] and with modifications as indicated elsewhere [33]. In short, hiPSCs of the stem cell line Ukki011-A were seeded at a density of 5 × 10^3^ cells cm^−2^ on day -3 on Matrigel-coated (Corning, Amsterdam, The Netherlands) 6-well plates and cultivated in mTeSR1 medium (Stem Cell Technologies, Vancouver, BC, Canada) until day 0, and then the medium was changed to unconditioned medium (DMEM/F12 with 20% knock-out serum replacement, 1% non-essential amino acids, 0.5% GlutaMAX and 0.1 µM 2-mercaptoethanol) from day 0 until day 5. On day 6, the cells were switched to endothelial medium with growth factors (heSFM with 1% platelet-poor plasma-derived human serum (PDS; SigmaAldrich, Taufkirchen, Germany), 20 ng mL^−1^ bFGF and 10 µM retinoic acid), and on day 8, they were sub-cultivated by dissociating them with Accutase for 35 min 37 °C. The dissociated cells were seeded onto membrane inserts (VWR, Darmstadt, Germany) pre-coated with 400 µg mL^−1^ human collagen IV (SigmaAldrich, Taufkirchen, Germany) and 100 µg mL^−1^ fibronectin (SigmaAldrich, Taufkirchen, Germany) in 0.05% acetic acid at a density of 1.0 × 10^6^ cells mL^−1^ in endothelial medium with growth factors. On day 9, the medium was changed to different media on both sides of the membrane—on the apical side, to enriched endothelial medium (heSFM with 5% PDS), and on the basolateral side, to heSFM.

#### 2.2.3. Cytotoxicity Testing

hBMECs were seeded at a density of 1.5 × 10^5^ cells cm^−2^ in 96-well plates. Lyophilised nanoparticles, both dopamine-containing and control nanoparticles without dopamine, were reconstituted in deionised water to an initial concentration of 5 mg mL^−1^ and shaken at 700 rpm and 4 °C for 30 min to allow the poloxamer 188 from the stabilizing agent to adhere to the nanoparticles. Afterwards, the nanoparticles were mixed 1:1 (*v*/*v*) with 10% FCS in PBS and agitated again at 700 rpm and 4 °C for 30 min to allow the serum proteins from the FCS to adhere to the coated nanoparticles. The incubated nanoparticles were diluted in cell culture medium in serial dilutions to cover a concentration range of 2.0 to 0.03 mg mL^−1^. Freshly prepared dopamine solution in cell culture medium was used as a control. Twenty-four hours after seeding, the prepared dilutions were applied to the cells and incubated for 4 h or 24 h at 37 °C, 5% CO_2_ and 90% relative humidity. At the end of the incubation period, the media either contained free dopamine or the dopamine contained in the nanoparticles was removed and fresh culture medium containing 10% WST-1 reagent (SigmaAldrich, Taufkirchen, Germany) was added. The plates were incubated at 37 °C, 5% CO_2_ and 90% relative humidity until a change in colour was visible. Identical incubation times were used in all experiments. Then, the absorbance was measured at 450 nm and at a reference wavelength of 690 nm with a Tecan infinite 200 plate reader (Tecan Group Ltd., Männedorf, Switzerland).

#### 2.2.4. Binding and Uptake Studies

bEnd.3 cells were seeded at a density of 1.25 × 10^4^ cells cm^−2^ in 12-well plates, proliferating LUHMES cells at a density of 2.5 × 10^4^ cells cm^−2^ and differentiated LUHMES cells at 7.5 × 10^4^ cells cm^−2^. Lyophilised nanoparticles were reconstituted as described earlier and diluted to a concentration of 0.5 mg mL^−1^ with pre-warmed bEnd.3 or LUHMES media as appropriate for each cell line, and the medium in the 12-well plates was exchanged for the nanoparticle suspension 48 h after seeding. The nanoparticles were incubated for 4 h at 37 °C, 5% CO_2_ and 90% relative humidity. Then, the nanoparticle-containing medium was removed, and the cells were washed three times with PBS and then collected from the wells by trypsin/EDTA incubation for 5 min. Following this, the cells were transferred to fresh medium and centrifuged for 4 min at 200× *g*, the supernatant was removed and the cell pellet was resuspended in 4% PFA before being measured using a FACSCalibur instrument (BD Biosciences, Heidelberg, Germany). A total of 10,000 cells per sample were measured, and the presence of nanoparticles in the cells was detected using the fluorescence signal of the Lumogen Red 305 dye contained in the particles.

#### 2.2.5. Transport Studies

Transport studies were performed on day 10 of cultivation of the hiPSC-derived hBCECs. Here, lyophilised nanoparticles were reconstituted as described earlier, diluted to a concentration of 2.5 mg mL^−1^ with 10% FCS in PBS, incubated on a rotating shaker at 700 rpm for 30 min and then applied to the blood-representing side of the model system at a concentration of 250 µg mL^−1^. After 4 h incubation at 37 °C, 5% CO_2_ and 90% relative humidity in an incubator, samples were taken from the apical and basolateral side of the membrane and stored at −20 °C until their preparation for the dopamine quantification. Trans-endothelial electrical resistance (TEER) values were monitored before and during nanoparticle incubation using the cellZscope device (nanoAnalytics, Münster, Germany) with hourly measurements to ensure the continued integrity of the hBCEC cell layer during the incubation period.

#### 2.2.6. Dopamine Quantification

Dopamine was quantified using an HPLC-MS technique. To prepare the samples, 10 µL of nanoparticle suspension or 50 µL of cell culture medium from the experiments were spiked with 10 µL dopamine-d4 (150 ng mL^−1^; SigmaAldrich, Taufkirchen, Germany) as an internal standard, mixed with 950 µL acetonitrile (ACN; LC/MS grade, Fisher Scientific, Schwerte, Germany) acidified with 0.2% heptafluor butyric acid (HFBA; LC/MS grade, Fisher Scientific, Schwerte, Germany), vortexed for 1 min and then sonicated for 10 min to achieve the complete destruction of the nanoparticles as well as the release of the contained dopamine. Then, 200 µL of water (LC/MS grade, Fisher Scientific, Schwerte, Germany) acidified with 0.2% HFBA was added to provide a better solvent for the dopamine. Next, the suspension was centrifuged for 5 min at 14,000× *g* and 4 °C to sediment any polymer chains, proteins or cellular debris. An 800 µL aliquot of the supernatant was used directly for HPLC-MS analysis. The measurements were performed on an Agilent 1260 Inifinity (Agilent technologies, Waldbronn, Germany) with a diode array detector coupled to a Q Exactive Focus mass spectrometer (Thermo Fisher Scientific, Dreieich, Germany). A 3 µL sample was injected per run and separated on a Accucore™ HILIC column (2.6 µm particle size, 3 mm × 100 mm) using 85% ACN with 0.2% HFBA and 15% water with 0.2% HFBA as the solvent at a solvent rate of 1 mL min^−1^. The column was heated to 40 °C, and each run was completed in 6 min. At the mass spectrometer, samples were exposed to an ionisation energy of 4 kV for positive ionisation before entering the capillary heated to 320 °C. Full MS-SIM spectra were recorded in an *m*/*z* range of 133.4 to 2000 for analysis. Dopamine peaks in a size range of 154.084 to 154.089 and dopamine-d4 peaks in a size range of 158.108 to 158.113 were isolated, and the area under the curve used for quantification in relation to a standard curve.

### 2.3. Statistical Analysis

All statistical data presented were calculated from at least three independent experiments; in this context, this means at least three different batches of nanoparticles or three independent cell culture experiments. For the nanoparticles, each batch produced consisted of one batch of nanoparticles containing dopamine and one batch without dopamine. Cell culture experiments were performed with relevant controls and with at least three technical replicates per parameter in each individual experiment.

## 3. Results

The dopamine-loaded PLGA nanoparticles were produced based on the method reported by Pahuja et al. [13], with modifications to optimise dopamine loading by modifying the first watery phase and the introduction of the primary emulsion into the second watery phase [14]. The resulting nanoparticles were characterised by determining their hydrodynamic diameter, PDI, zeta potential and dopamine loading. For each batch of dopamine-loaded nanoparticles, a comparable batch without dopamine was also produced. As can be seen in Table 1, the nanoparticles containing dopamine had a diameter of 198.1 ± 3.4 nm, while those without dopamine were slightly larger at 207.5 ± 5.4 nm. The PDIs of both particle batches were comparable, with 0.03 ± 0.01 and 0.04 ± 0.02 for particles with or without dopamine, respectively. The zeta potential was negative, with −29.1 ± 1.7 mV for dopamine-loaded and −33.8 ± 2.5 mV for dopamine-free nanoparticles. Dopamine could only be detected in the nanoparticles intended to contain it, and the loading was 4.16 ± 0.39 µg dopamine per mg nanoparticle.

**Figure 1:**
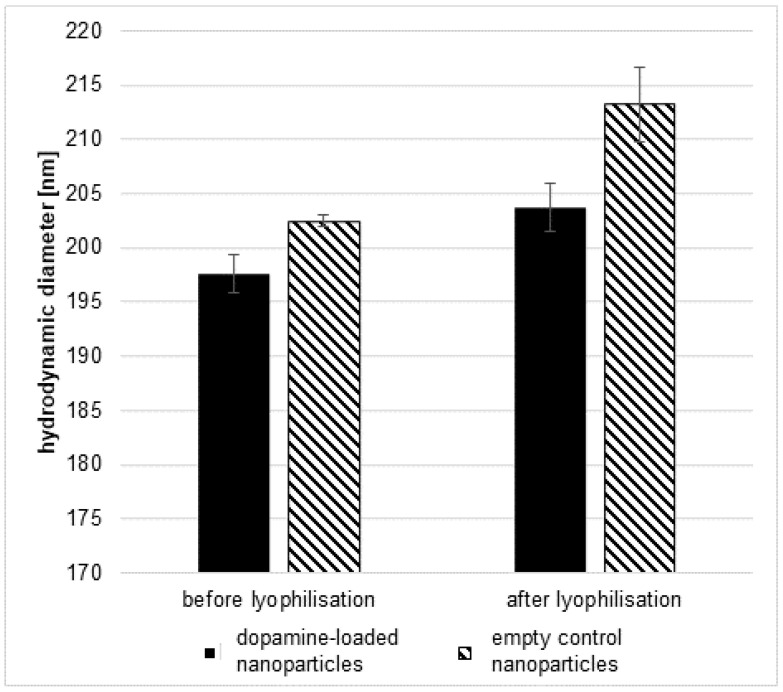
Hydrodynamic diameter of dopamine-loaded (black) and unloaded control (lined) nanoparticles before and after lyophilisation, measured using the zetasizer ZS. The mean ± S.D was determined from at least 3 independent batches of nanoparticles.

To allow the produced nanoparticles to cross the blood–brain barrier in order to transport the contained dopamine to the brain, surface modification was necessary. As has been described before [34], coating the surface with a surfactant such as poloxamer 188 allows PLGA nanoparticles to cross the blood–brain barrier. As the nanoparticles are intended to be used in a therapeutic context, mid- to long-term storage stability must be considered. For this purpose, the nanoparticles were freeze-dried and stored under the exclusion of daylight in airtight glass vials at 4 °C until immediately prior to use. Experimental results showed a 0.5 ± 0.04% water content in the samples, indicating a successful lyophilisation that allowed storage and easier transport. As a stabilising agent for the lyophilisation process, poloxamer 188 was used. Using a solution of the same substance used for coating as the stabiliser for lyophilisation has a definite advantage. The uncoated nanoparticles can be safely lyophilised and are exposed to the intended coating agent during reconstitution, eliminating the need for an additional coating step. Successful coating was shown by an increase in the hydrodynamic diameter of the nanoparticles after lyophilisation and reconstitution in comparison with its values before lyophilisation. The size increase was 6.1 ± 2.4 nm for dopamine-loaded and 10.7 ± 3.4 nm for unloaded nanoparticles (Figure 1). Differences in the size of the nanoparticles between dopamine-loaded and unloaded nanoparticles are due to the influence of dopamine on the production process of the nanoparticle as it approaches the average hydrodynamic diameter.

**Figure 2:**
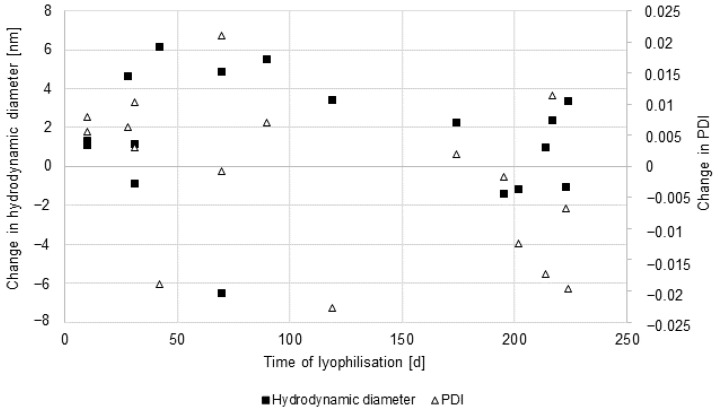
Storage stability of lyophilised nanoparticles. Nanoparticles were stored in lyophilised form at 4 °C before reconstitution. Hydrodynamic diameter (black square) and PDI (white triangle) were determined using the zetasizer ZS.

To evaluate the stability of the nanoparticles while stored in lyophilised form, different batches of nanoparticles were reconstituted after different storage times. Storage times ranged from 10 to 224 days. Differences in the hydrodynamic diameter and the PDI are shown in Figure 2.

The change in hydrodynamic diameter was in a range of less than 8 nm, and there was no discernible trend in relation to storage time. The PDI changed within a range of less than 0.025 with no discernible trend as well. This indicates that the nanoparticles were stable under the used storage conditions.

Prior to the in vitro transport study, all reconstituted nanoparticle suspensions were again characterised with regard to their key parameters size, size distribution, hydrodynamic diameter and zeta potential. The results are summarised in Table 2. 

**Figure 3:**
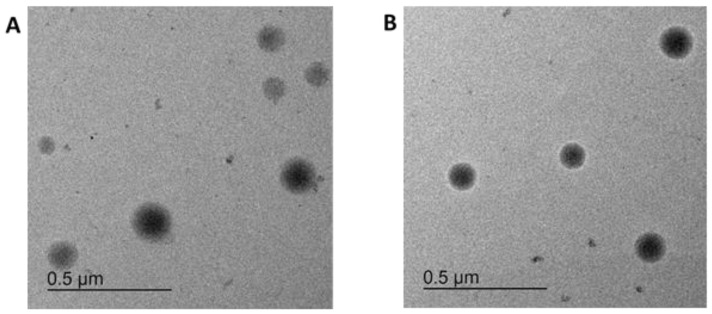
Transmission electron micrographs of (**A**) particles loaded with dopamine and Lumogen Red 305 dye and (**B**) particles loaded only with Lumogen Red 305 dye.

The TEM investigation showed single, round-shaped particles of varying sizes (Figure 3). 

The mean particle diameters determined from TEM micrographs were 203 ± 44 nm (particles loaded with dopamine and Lumogen Red 305 dye) and 167 ± 63 nm (particles loaded with Lumogen Red 305 dye), indicating a wide particle-size distribution. The nanoparticle tracking analysis indicated hydrodynamic diameters of 187 ± 55 nm (particles loaded with dopamine and Lumogen Red 305 dye) and 184 ± 52 nm (particles loaded with Lumogen Red 305 dye), demonstrating the same hydrodynamic size for both particle types with a wide particle distribution. The similar magnitudes of the primary size (analysed by TEM) and hydrodynamic size (determined by NTA) indicate that there is hardly any particle aggregation when the nanoparticles are suspended in water. As a further parameter, the surface charge was analysed by zeta potential measurement of the particles in water. The values of −4.6 ± 0.5 mV (particles loaded with dopamine and Lumogen Red 305 dye) and −4.7 ± 0.5 mV (particles loaded with Lumogen Red 305 dye) represent a close-to-neutral surface charge, which indicates an effective particle coating with uncharged poloxamer 188. The slightly negative charge could result from free carboxylate end groups of the PLGA polymer present on the particle surface.

To evaluate the stability of the particles systems, the leaching of the Lumogen Red 305 dye from the nanoparticles was evaluated as measure of overall stability, as an equal distribution of the dye within the nanoparticle structure was assumed based on its inclusion in the same phase as the polymer itself during the nanoparticle production. Neither the nanoparticles containing dopamine and Lumogen Red 305 nor the nanoparticles containing only Lumogen Red 305 showed a significant change in the amount of Lumogen Red 305 found in the filtrate in comparison to the overall fluorescence of the samples, indicating that the nanoparticles remained stable in regard to the Lumogen Red 305 content during the investigated time period. The results are shown in Table 3.

**Figure 4:**
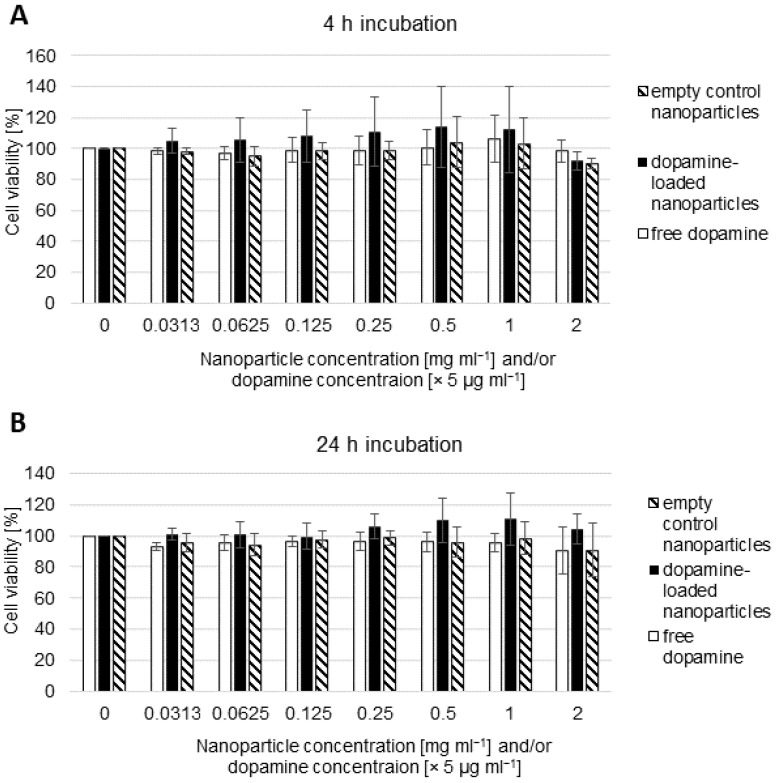
Cell viability of hBMECs after (**A**) 4 h or (**B**) 24 h exposure to empty control nanoparticles (hatched bars), dopamine-loaded nanoparticles (black) or free dopamine (white). Viability was measured using the WST-1 reagent. The mean ± S.D. was calculated from three independent experiments with different batches of nanoparticles.

The produced nanoparticles were tested for their cytotoxicity and, therefore, the ability to mask the effect of the contained dopamine (Figure 4). At the tested concentration ranges of 0.03 to 3 mg mL^−1^ nanoparticles or 0.16 to 10 µg mL^−1^ dopamine, no significant decrease in cellular viability was observed. Neither the unloaded nanoparticles themselves nor the dopamine-loaded version caused a cytotoxic effect in these endothelial cells representing the blood–brain barrier within the concentration range used in the experiments.

**Figure 5:**
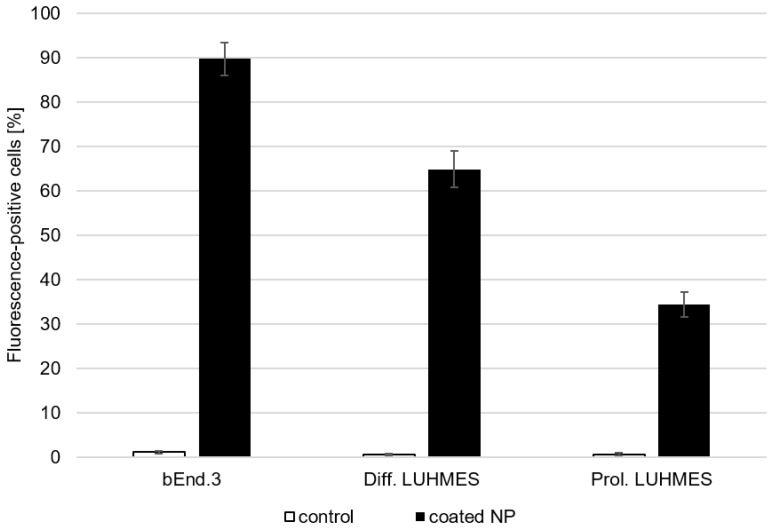
Flow cytometry analysis of bEnd.3 cells, differentiated LUHMES cells and proliferating LUHMES cells after 4 h of incubation with poloxamer 188-coated, dopamine-loaded nanoparticles with Lumogen Red 503 dye (black) in comparison with control cells without nanoparticle incubation (white). Nanoparticles were coated during reconstitution from lyophilisates and pre-incubated with 10% FCS in PBS for 30 min before being added to the cells. The mean ± S.D. was calculated from three independent experiments with different batches of nanoparticles.

Cellular binding and uptake studies were performed on cell lines representing the targets of the dopamine delivery: in this case, cells representing the blood–brain barrier and dopaminergic neurons. bEnd.3 cells were used to represent the blood–brain barrier, as they express the appropriate blood–brain-barrier markers while being much less time consuming to produce in sufficient amounts for the experiments [35]. The LUHMES cell line was used in its undifferentiated form as unspecific neuronal precursor cells as well as in its differentiated form as dopaminergic neurons [31] to evaluate the specificity of the uptake in these neuronal cells. As shown in Figure 5, 89.2% of the bEnd.3, 64.9% of the differentiated LUHMES cells and 34.4% of the proliferating LUHMES cells took up the poloxamer-188-coated nanoparticles. Therefore, the uptake in specific dopaminergic neurons is notably higher than in unspecific neuronal cells.

**Figure 6:**
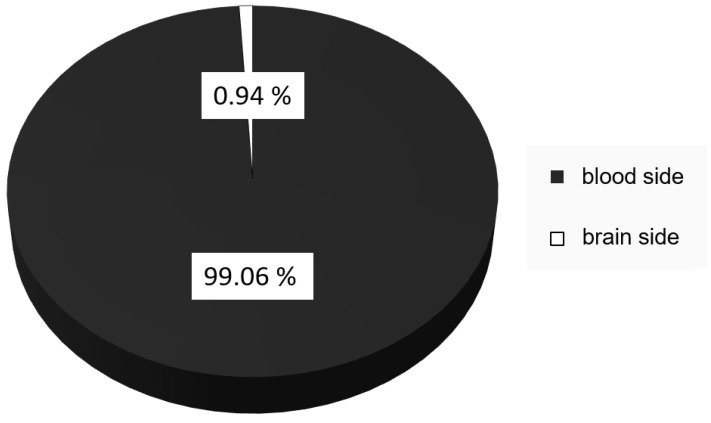
Transport of dopamine across an hiPSC-derived blood–brain barrier model. Lyophilised nanoparticles were reconstituted and re-incubated in 10% FCS in PBS before being added to the apical side of the BBB model. After 4 h of incubation, samples were collected from the blood (dark grey) and brain (white) sides of the BBB, and the dopamine content was determined by HPLC-MS. The mean ± S.D. was calculated from three independent experiments with different batches of nanoparticles.

The transport of the nanoparticles across the blood–brain barrier was determined using a three-dimensional blood–brain barrier model with brain capillary endothelial cells differentiated from hiPSCs of a healthy donor. The hBCECs were cultivated on a porous membrane, with the apical compartment representing the blood side of the barrier and the basolateral compartment representing the brain side. Nanoparticles were applied to the blood-representing side, and transport was measured indirectly by determining the amount of dopamine found on the brain-representing side of the model. Only 0.94% of the applied nanoparticulate dopamine was found on the brain side after 4 h of incubation, while the remaining 99.06% remained on the blood side (Figure 6).

## 4. Discussion

The successful transport of dopamine across this hiPSC-derived blood–brain-barrier model system supports the information reported by Pahuja et al. [13] in their animal experiments. While the transported amount of 0.94% after 4 h incubation over a surface of 1.1 cm^2^ seems much lower than the uptake reported in Pahuja’s paper, it is necessary to consider that the time course of nanoparticle uptake in vivo is much faster than that achieved in in vitro models, as could be shown by Zensi et al. [26]; while the investigated nanoparticle uptake could be shown after 4 h in vitro, in the comparable in vivo model, they could already be detected in the brain tissue after 15 min. Additionally, the surface area of the 1.1 cm^2^ inserts is much lower than the surface area of the capillaries in the brain, especially when compared with the human brain, with its surface area of approximately 12 m^2^ [36]. The transport of nearly 1% has to be considered a positive result and indicative of successful transport, especially considering that the human brain represents approximately 1% of the mass of the human body and that a concentration of 1% therefore represents an even systemic distribution [37]. In the context of 3R, this also indicates that a number of animal experiments could potentially be replaced by advanced in vitro models, but some additional evaluations, specifically to better correlate the time-dependent transport effect, should be considered.

In a more detailed evaluation of the present study, the modified particle-preparation strategy used in this study provided nanoparticles with reproducible characteristics in size, PDI, zeta potential and dopamine loading. For the encapsulation of hydrophilic compounds like dopamine, the use of double-emulsion techniques has been discussed as the method of choice for a number of years [13,14,38], as such strategies have proven to be superior to simpler processes such as nanoprecipitation [39,40]. Due to the staggered emulsion of the hydrophilic dopamine when dissolved in an initial aqueous phase into the organic phase as the first emulsion, the dopamine is encouraged to remain in hydrophilic regions of this initial emulsion when a larger quantity of a second water-based phase is introduced in a second step to form a new emulsion. While the amount of hydrophilic environment around the first emulsion at the introduction of the second aqueous phase will draw the majority of the dissolved dopamine, the amount retained in the produced nanoparticles is still increased when compared to with the dopamine that would have been retained in the organic phase without introducing the first emulsion. There is debate as to whether the encapsulation of small hydrophilic compounds such as dopamine would be further improved by adjusting the organic phase to be more miscible with the aqueous phases [38] while also improving characteristics such as size and PDI. Here, we showed that the use of an established solvent evaporation method produced reliable nanoparticles of sufficiently small size and biocompatibility, and a switch to a solvent diffusion process was not necessary. Still, the decision for or against such approaches should be investigated on a case-by-case basis depending on the targeted results and encapsulation amounts.

Overcoming the challenge of encapsulating dopamine in nanoparticles is only the first step to providing an option for dopamine delivery to the brain. The produced nanoparticles also have to reach the brain, where the dopamine is needed. Targeting nanoparticles is possible in a wide range of processes, depending on the intended target region. Reaching the brain is one of the most challenging endeavours due to the high selectivity of the blood–brain barrier [41]. The most promising avenues involve surface modifications targeting the specific transport mechanisms most prevalent in the brain capillary endothelial cells, be it receptor- or transporter-mediated transport [42]. To achieve this, specific substances, peptides or proteins that are recognised by these processes have to be present on the nanoparticle surface. While the direct linking of these substances to the particle surface is widely used [22,43,44], simpler surface coatings are also able to attract plasma proteins, which will then facilitate the uptake at the blood–brain barrier [34,45]. This shortens the nanoparticle production process and thus allows fewer possibilities to lose some amount of the encapsulated dopamine at each of the steps. The nanoparticles used here, with a simple surface coating of poloxamer 188 introduced during the lyophilisation process, present the least time-consuming and easiest method of targeting the nanoparticles to the brain. The success of this strategy has been shown by the excellent uptake values in the bEnd.3 cells as a simple model system and the still high uptake even in the dopaminergic neurons derived from differentiated LUHMES cells. Successful transport of the contained dopamine could be shown in an advanced blood–brain barrier model using brain capillary endothelial cells derived from hiPS cells, as discussed above. 

To ensure that any perceived transport behaviour is not based on barrier degradation processes, cytotoxicity assays were also performed for the dopamine-loaded nanoparticles in comparison to unbound dopamine. The hBMEC human brain microvessel cell line showed no cytotoxic reaction to the quantity of nanoparticles in the experiments when used at 25 µg mL^−1^ and even higher concentrations. Therefore, the possibility of transport being made possible by barrier degradation can be excluded, and the amount of dopamine found on the basolateral side is from actively crossing the blood–brain barrier.

We have demonstrated the successful use of an advanced in vitro blood–brain barrier model that supports the findings achieved in previous in vivo experiments [13]. Thus, as soon as reliable model systems are established and proven, initial experiments on nanoparticle uptake and transport should first be evaluated in an in vitro setting before utilising in vivo experiments to reduce the amount of animal experiments necessary in preclinical studies.

## Figures and Tables

**Table 1:** Characterisation of PLGA nanoparticles before freeze-drying. The hydrodynamic diameter, PDI and zeta potential were determined with the zetasizer ZS and the dopamine content by HPLC-MS analysis. The mean ± S.D. was calculated from at least three different batches of nanoparticles.

	Hydrodynamic Diameter [nm]	PDI	Zeta Potential [mV]	Dopamine Content [µg/mg NP]
with dopamine	198.1 ± 3.4	0.03 ± 0.01	−29.1 ± 1.7	4.1 ± 0.4
without dopamine	207.5 ± 5.5	0.04 ± 0.02	−33.8 ± 2.5	—

**Table 2:** Physicochemical properties of reconstituted particles: primary particle size (analysed by transmission electron microscopy, TEM), hydrodynamic diameter (analysed by nanoparticle tracking analysis, NTA) and zeta potential of particles in water (supplemented with 0.01 M KCl as the background electrolyte).

Particle Type	TEM	NTA	Zeta Potential
particle with dopamine and with Lumogen Red 305 dye	203 ± 44 nm	187 ± 55 nm	−5 ± 1 mV
particle with Lumogen Red 305 dye	167 ± 63 nm	184 ± 52 nm	−5 ± 1 mV

**Table 3:** Leaching of the Lumogen Red 305 dye from nanoparticles. Lyophilised nanoparticles were reconstituted and diluted in PBS. Dye leaching was determined 0 h and 24 h after reconstitution from a filtrate of the nanoparticles. Data are mean values from three independent centrifugation procedures.

Particle Type	Fluorescence of the Filtrate [%] at t = 0 h	Fluorescence of the Filtrate [%] at t = 24 h
particle with dopamine and with Lumogen Red 305 dye	4.8 ± 0.1	5.6 ± 3.6
particle with Lumogen Red 305 dye	6.2 ± 0.3	7.4 ± 2.7

## Data Availability

The data presented in this study are available on request from the corresponding author.
